# Robust trend tests for genetic association in case-control studies using family data

**DOI:** 10.1186/1471-2156-6-S1-S107

**Published:** 2005-12-30

**Authors:** Xin Tian, Jungnam Joo, Gang Zheng, Jing-Ping Lin

**Affiliations:** 1 Office of Biostatistics Research, National Heart, Lung and Blood Institute, 6701 Rockledge Dr., Bethesda, Maryland 20892, USA

## Abstract

We studied a trend test for genetic association between disease and the number of risk alleles using case-control data. When the data are sampled from families, this trend test can be adjusted to take into account the correlations among family members in complex pedigrees. However, the test depends on the scores based on the underlying genetic model and thus it may have substantial loss of power when the model is misspecified. Since the mode of inheritance will be unknown for complex diseases, we have developed two robust trend tests for case-control studies using family data. These robust tests have relatively good power for a class of possible genetic models. The trend tests and robust trend tests were applied to a dataset of Genetic Analysis Workshop 14 from the Collaborative Study on the Genetics of Alcoholism.

## Background

Testing for linkage disequilibrium or association provides a useful alternative to testing linkage for complex traits with relatively small genetic effects [1]. Among the tests for association between a candidate-gene and a disease within a case-control design, the Cochran-Armitage (CA) trend test [2,3] is preferable to the allele-based test and the Pearson's chi-squared test [4-6]. In such studies, cases and controls are usually independent random samples. Genotypes on each individual at markers in or near candidate genes are observed. For a marker with two alleles, the CA trend test can be used to test a linear trend between the disease and the number of the high-risk alleles at this marker.

Recently, there has been an increasing interest in statistical methods that evaluate association between genetic markers and disease status using family-based data [7,8]. This would allow data available from linkage studies to be efficiently used to test for association. Unlike the traditional case-control studies in which all individuals are unrelated, cases and controls drawn from family data are often correlated because these individuals are often biologically related. Consequently, the frequencies of the high-risk alleles at a marker locus will be increased among related individuals. This may affect the false positive rate (type I error) for the association test, compared to case-control design based on independent samples. Hence, any test of genetic association must account for the correlations among family members. Slager and Schaid [7] extended the original CA trend test to case-control studies with family data, in which they modeled the correlations among related cases or controls as functions of the probability of their marker alleles shared identically by descent (IBD). This method can be applied to complex family structures and it obtains different correlations for different types of relative pairs. Thus, it is more flexible than the method assuming a common correlation for each pair of relatives within a family. With this correlation adjusted, the resulting trend test in Slager and Schaid [7] is similar to the original one but it uses appropriate variance formulation. Note that this trend test uses different scores depending on assumptions of the underlying genetic models. In practice, because the genetic model is unknown for most, if not all, complex diseases, applying a trend test with one set of scores would result in loss of power if the genetic model is misspecified. Therefore, more robust tests have been proposed to protect against model uncertainty [9,10].

In this paper we study the two robust trend tests, the maximum test (MAX) and maximin efficiency robust test (MERT), in case-control design applied to family data. These two robust tests account for the correlated individuals and do not rely on the assumption of any particular genetic model. The performance of the robust trend tests and the extended CA trend test is compared by a simulation study. These tests are illustrated using a Genetic Analysis Workshop 14 dataset from the Collaborative Study on the Genetics of Alcoholism (COGA).

## Methods

### The trend tests

Consider data for a case-control study of genetic association as in Table 1. Assume a marker with two alleles: *N *and *M*, where *N *is a normal allele and *M *is an allele with high risk. Denote genotypes as *g*_0 _= *NN*, *g*_1 _= *NM*, and *g*_2 _= *MM*. Let the genotype frequencies for cases and controls to be *p*_*j *_and *q*_*j*_, *j *= 0, 1, 2, respectively, and . Hence, the null hypothesis of no association is to test *p*_*j *_= *q*_*j *_for each *j*.

Given the data, the CA trend test for association [4] between a disease and the marker is written as *Z*_*x *_= *U*(***x***)/(Var[*U*(**x**)])^1/2^, where , and ***x ***= (*x*_0_, *x*_1_, *x*_2_)' is a set of increasing scores (weights) assigned to the three genotypes (*g*_0_, *g*_1_, *g*_2_) *a priori *based on the underlying genetic model. Note that (*x*_0_, *x*_1_, *x*_2_)' can be reparameterized as (0, *x*,1)' with 0 ≤ *x *≤ 1. If cases and controls are from independent random samples, the counts (*r*_0_, *r*_1_, *r*_2_) and (*s*_0_, *s*_1_, *s*_2_) in Table 1 follow multinomial distributions *mul*(*R*; *p*_0_*, p*_1_, *p*_2_) and *mul*(*S*_*; *_*q*_0_, *q*_1_, *q*_2_), respectively. Under the null hypothesis, it can be shown that , and *Z*_*x *_asymptotically follows a standard normal distribution *N*(0, 1).

The null hypothesis *H*_0 _is rejected in favor of the alternative that *M *is the high risk allele associated with disease when *Z*_*x *_>*z*_1-*α*_, where *z*_1-*α *_is the upper 100(1 - *α*)^th ^percentile of *N*(0, 1). When it is not certain which allele is high-risk, *H*_0 _is rejected when |Z_x_| > z_1-*α*/2_.

However, since for case-control studies drawn from family data, cases and controls within the same family may be biologically related, Slager and Schaid [7] proposed the following method for estimating the variance to account for correlations among related cases or controls. Let ***y***_*i *_= (*y*_*i*0_, *y*_*i*1_, *y*_*i*2_)' be the genotype indicator vector for the *i*^th ^case, where *y*_*ij *_= 1 for the *i*^th ^case with genotype g_*j *_and *y_ij_*= 0 otherwise, *i *= 1, ..., *R*. Similarly, we use ***z***_*j *_for controls. Then **r **= (*r*_0_, *r*_1_, *r*_2_)' = , and **s** = (*s*_0_, *s*_1_, *s*_2_)' = . Furthermore, ***y***_*i *_and ***z***_*j *_follow the multinomial distributions *mul*(1; *p*_0_, *p*_1_, *p*_2_) and *mul*(1; *q*_0_, *q*_1_, *q*_2_), respectively. Let *φ *= *R*/*n*. The test statistic *U*(**x**) can also be written as *U*(**x**) = **x**'[(l - *φ*) **r **- *φ***s**]. Then,



where the variances and covariances can be calculated based on the multinomial distributions and IBD-sharing probabilities for pairs of the related individuals [7],

### Robust trend tests when the genetic model is unknown

Because for most complex diseases the underlying genetic model is unknown, we consider two robust trend tests [9,10], the MERT and the MAX in the case-control study, where the *cases *and controls may be related. Note that for the special case in which cases and controls are independent random samples, the tests have been studied by Friedlin et al. [10].

Suppose we have a family of trend test statistics *Z*_*i*_corresponding to different genetic models. The first robust test, MERT, can be written as a linear combination of the two test statistics with minimum correlation *ρ*_0_. Denoting these two tests as {*Z*_*s*_, *Z*_*t*_}, then MERT is written as *Z*_MERT_=(*Z*_*s *_+ *Z*_*t*_)/{2(1 + *ρ*_0_)}^1/2^, which asymptotically follows a standard normal distribution. The second robust trend test, MAX, can be defined as *Z*_MAX_=max(*Z*_*s*_, Z_MERT_, *Z*_*t*_) for a one-sided test, and *Z*_MAX _= max(|*Z*_*s*_|, |Z_MERT_|, |*Z*_*t*_|) for a two-sided alternative, where *Z*_MERT _is chosen as the "middle" test because it has equal correlations with *Z*_*s *_and *Z*_*t*_. MAX is more powerful than MERT when *ρ*_0 _is small, and the two tests have similar power when the minimum correlation is relatively large (e.g., *ρ*_0 _≥ 0.75) [11].

For case-control studies drawn from family data, we can derive the correlations for the trend tests defined in the previous section. Let the variance-covariance matrix . Then the correlation between any two test statistics can be obtained



where **x**_0 _and **x**_1_are two sets of scores used for two different genetic models.

To test for association between a marker and disease status, the optimal scores for the recessive, additive, and dominant models are *x *= 0, 1/2, and 1 in **x **= (0, *x*, 1)' [12]. Based on the prior scientific knowledge, other possible choices of genetic models can also be assumed, which leads to different trend tests. The correlation of any two tests can then be calculated to determine the pair of tests with minimum correlation, so the MERT test can be performed. To apply the MAX test, the critical value and the *p*-value are obtained from simulation.

### The trend tests with multiple alleles

The above trend tests *Z*_x _can be extended to test the association with a multiallelic marker in a case-control study [7]. For a marker with *K *different alleles, there are *m *= *K*(*K *+ 1)/2 possible genotypes and we can obtain a case-control table with *r*_*i *_and *s*_*i*_, *i *= 1, ..., *m*, similar to Table 1. The trend test statistic can be written as a (*K-*1) × 1 vector, *U *= *U*(**X**) = **X**' [(1-*φ*)**r **-*φ***s**], where **X **is a *m *× (*K *- 1) matrix with the *j*^th ^column, **x**_*j*_, as a score vector for the m genotypes corresponding to the *j*^th ^allele, and Var(*U*)= **X'**∑**X **can be obtained similarly as in the previous section to adjust for correlations among family members. To test the association with this marker, Slager and Schaid [7] proposed to use the statistic *U'*[Var(*U*)]^-1 ^*U *as it asymptotically follows a chi-squared distribution with (*K *- 1) degrees of freedom.

Here, we can apply MERT and MAX as alternatives to this chi-squared test. Corresponding to the *j*^th ^allele, the *j*^th ^element *of U *is *U*_*j *_= **x'**_*j*_[(1-*φ*)**r**-*φ***s**], and we have  = Var(*U_j_*) = **x'***_j_*∑**x**_*j *_and . Then the trend test for each allele, Z_*j *_= U_*j*_/*σ*_*j*_, *j *= 1,..., (*K *- 1), and the correlation for any two tests can be obtained. Hence, for the family of trend tests, MERT and MAX can be used to test for association with a multi-allelic marker.

## Results

### A simulation study

To illustrate the robustness of the statistics, MERT, and MAX, and to compare their performance with individual trend tests for given models, we simulated the case-control datasets and computed the empirical powers for all the tests under three genetic models: the recessive, additive and dominant models.

The simulations were based on the assumptions that the disease prevalence *K *= 0.1 and the allele frequency p = 0.3 with 20,000 replications. To facilitate the calculation, each case-control dataset included 160 cases generated as 80 sib-pairs drawn from 80 different families, and 160 controls as unrelated random samples. It can be shown that the probabilities of 0, 1, 2, alleles shared IBD are 1/4, 1/2, and 1/4 for the sib-pairs when parents' genotype information was unknown. Assuming these IBD probabilities, the variance of the trend test was adjusted for the correlations among related cases. Let the genotype relative risks *RR*_1 _= *f*_1_/*f*_0 _and *RR*_2 _= *f*_2_/*f*_0_, where *f*_0_, *f*_1_, and *f*_2 _are penetrances for genotypes *g*_0_, *g*_1_, and *g*_2_. Thus, equivalently, the null hypothesis *H*_0 _can be written as *RR*_1 _= *RR*_2 _= 1. The alternative hypothesis can be specified by varying *RR*_1 _and *RR*_2_.

Table 2 displays the empirical powers of the trend tests and the robust tests, MERT and MAX. The relative risks *RR*_1 _and *RR*_2 _were chosen so that a particular trend test had about 80% power for each given model. When the true underlying model was recessive inheritance and the corresponding optimal test *Z*_(*x *= 0) _had power of 80%, the tests *Z*_(*x *= 1/2) _and *Z*_(*x *= 1) _only had power of 62% and 26%, respectively. However, the test *Z*_(*x *= 0) _was underpowered when the true model was dominant or additive. Compared to these trend tests, the MERT and MAX tests had relatively good powers for all the three models.

### Application

The COGA data consist of 1,614 individuals from 143 families, with alcoholism diagnosis, microsatellite, and single-nucleotide polymorphism (SNP) marker information. The preliminary genome scan by linkage analysis using the microsatellite data suggested that *ADH3 *of chromosome 4 may be an alcoholism susceptibility gene. Without adjusting for family structure, a logistic regression with backward selection of SNPs from the Illumina dataset near the *ADH *genes indicated that SNP marker rs1037475 was a significant predictor. Here we applied the association tests to case-control data using the ALDX1 diagnosis of "affected" and "purely unaffected" status to define case status and genotypes for this SNP marker. Table 3 presents the data including cases from 143 families and controls from 111 families.

**Figure 1:**
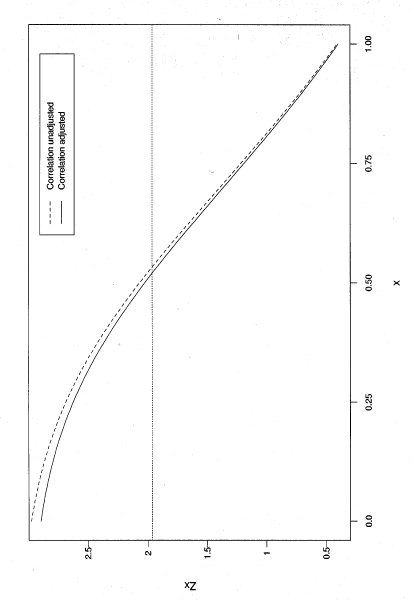
Plot of the trend tests Z_x _versus scores *x*.

Results of trend tests for the data in Table 3 with or without adjusting for the family-based correlations are shown in Figure 1. For individuals from the same family, their shared alleles IBD probabilities were calculated using software GENEHUNTER [13], and the correlations and the adjusted variances of the test statistics were obtained. We then applied the two-sided trend tests under recessive, additive, and dominant models, corresponding to the scores *x = *0, 1/2, and 1. The tests showed significant association under both the recessive and additive model assumption (*Z*_(*x *= 0) _= 2.89, *p *= 0.004; *Z*_(*x *= 1/2) _= 2.02, *p *= 0.043), but it failed to show any significant result assuming a dominant model (*Z*_(*x *= 1) _= 0.40, *p *= 0.69). Note that after adjusting for the correlations among family members, standard errors were larger, resulting in smaller test statistics *Z*_*x *_and thus larger *p*-values compared to the tests without adjusting for the correlations (see Figure 1).

Figure 1 also shows the trend test results depend on the scores *x *= (0, *x*, 1) for the underlying genetic models. The trend tests *Z*_*x *_with 0 ≤ *x *≤ 1 correspond to different models, where the statistics *Z*_*x *_above the horizontal dotted line are significant. Due to the uncertainty about the mode of inheritance, different conclusions could be reached and using any single trend test may result in significant loss of power when the model is misspecified. Therefore, we also applied the two robust tests to these data. Given the tests for the recessive, additive, and dominant models, the pair-wise correlations were calculated as Corr(*Z*_(*x *= 0)_, *Z*_(*x *= 1)_) = 0.334, Corr(*Z*_(*x *= 0)_, *Z*_(*x *= 1/2)_) = 0.818, and Corr(*Z*_(*x *= 1/2)_, *Z*_(*x *= 1) _= 0.813. Then we obtained *Z*_MERT _= (2.89 + 0.40)/{2(1 + 0.334)}^1/2 ^= 2.01 with *p*-value = 0.044. By simulations with 1,000,000 replications, the empirical *p*-value for *Z*_MAX _= 2.89 was *p *= 0.009. In this example, because the correlation between the test statistics under the recessive and dominant models is small, MAX appears to be more powerful than MERT to detect associations between disease status and a marker. Both robust trend tests showed significant association between this SNP marker and alcoholism.

## Conclusion

In this paper, we applied the trend tests of genetic association to case-control studies drawn from the COGA families. Although the significant results under the recessive, additive, and dominant models were similar for this example, the tests ignoring the correlations among family members would have yielded large false-positive rates and moreover, unadjusted tests would not be valid.

We have also studied two robust trend tests, MERT and MAX, for case-control studies with family data. When the genetic model is unknown, these robust tests based on a family of possible genetic models tend to be more conservative against model misspecification. Although we have focused on the examples and models for genetic association, these results hold generally for trend tests of association with correlated cases or controls when the exposure variables have some natural ordering.

## Abbreviations

CA: Cochran-Armitage

COGA: Collaborative Study on the Genetics of Alcoholism

IBD: Identical by descent

MAX: Maximum test

MERT: Maximin efficiency robust test

SNP: Single-nucleotide polymorphism

## Authors' contributions

XT involved in the design of the study and statistical analysis, and drafted the manuscript. JJ, GZ, and JPL participated in its design and performed the statistical analysis. All authors read and approved the final manuscript.

## Figures and Tables

**Table 1:** The data in a case-control study

Status	*NN*	*NM*	*MM*	Total
Case	*r*_0_	*r*_1_	*r*_2_	*R*
Control	*s*_0_	*s*_1_	*s*_2_	*S*

Total	*n*_0_	*n*_1_	*n*_2_	*n*

**Table 2:** Empirical powers of trend tests and robust trend tests

Model	(*RR*_1_, *RR*_2_)	***Z***_(*x *= 0)_	***Z***_(*x *= 1/2)_	***Z***_(*x *= 1)_	MERT	MAX
Null	(1,1)	0.05	0.05	0.05	0.05	0.05
Recessive	(1,2.6)	0.80	0.62	0.26	0.70	0.76
Additive	(1.2,2.4)	0.44	0.80	0.73	0.77	0.75
Dominant	(1.9,1.9)	0.18	0.72	0.80	0.64	0.73

**Table 3:** A case-control dataset from the COGA study

Status	*NN*	*NM*	*MM*	Total
Case	153	278	178	609
Control	69	141	51	261

Total	222	419	229	870
